# "Do-Nothings" and the College. A Striking Condemnation of Tactics

**Published:** 1919-03-15

**Authors:** M. P. Ferrier


### "Do-Nothings" and the College. A Striking Condemnation of Tactics.

*To the Editor of* The Hospital.

Sir,—It is strange the Central Committee do not realise that their many contentions against the work that is being done on behalf of nurses by the College of Nursing, limited by guarantee, is really at the root of all these extraordinary side issues we read of. Regarding the work on the improvement of the pay and working hours of nurses which is being done, this takes time, but it seems that the object of some members of the Central Committee is to raise difficulties and also to create distrust. Some lack of imagination is therefore shown by them. Surely a few members of this blind to nurses' interests Committee must know how inconsistent is their attitude, and that it would be more consistent with their any work genuinely done on behalf of the nursing profession if they were to unite their efforts with those of the College and thus put an end to all further controversy.

Yours truly,

M. P. Ferrier.

